# Study on the isoprene-producing co-culture system of *Synechococcus elongates*–*Escherichia coli* through omics analysis

**DOI:** 10.1186/s12934-020-01498-8

**Published:** 2021-01-07

**Authors:** Hui Liu, Yujin Cao, Jing Guo, Xin Xu, Qi Long, Lili Song, Mo Xian

**Affiliations:** CAS Key Laboratory of Bio-Based Materials, Qingdao Institute of Bioenergy and Bioprocess Technology, Chinese Academy of Sciences, Qingdao, 266101 China

**Keywords:** Co-culture, *Synechococcus elongates*, *Escherichia coli*, Isoprene, Continuous microbial production

## Abstract

**Background:**

The majority of microbial fermentations are currently performed in the batch or fed-batch manner with the high process complexity and huge water consumption. The continuous microbial production can contribute to the green sustainable development of the fermentation industry. The co-culture systems of photo-autotrophic and heterotrophic species can play important roles in establishing the continuous fermentation mode for the bio-based chemicals production.

**Results:**

In the present paper, the co-culture system of *Synechococcus elongates*–*Escherichia coli* was established and put into operation stably for isoprene production. Compared with the axenic culture, the fermentation period of time was extended from 100 to 400 h in the co-culture and the isoprene production was increased to eightfold. For in depth understanding this novel system, the differential omics profiles were analyzed. The responses of BL21(DE3) to *S. elongatus* PCC 7942 were triggered by the oxidative pressure through the Fenton reaction and all these changes were linked with one another at different spatial and temporal scales. The oxidative stress mitigation pathways might contribute to the long-lasting fermentation process. The performance of this co-culture system can be further improved according to the fundamental rules discovered by the omics analysis.

**Conclusions:**

The isoprene-producing co-culture system of *S. elongates*–*E. coli* was established and then analyzed by the omics methods. This study on the co-culture system of the model *S. elongates*–*E. coli* is of significance to reveal the common interactions between photo-autotrophic and heterotrophic species without natural symbiotic relation, which could provide the scientific basis for rational design of microbial community.

## Background

With the depletion of the nonrenewable petrochemical resources, many biofuels and platform chemicals have been industrially produced by fermentation to substitute the petroleum-based counterparts. However, the majority of microbial fermentations are currently performed within a short period of time in the batch or fed-batch manner, which means the high process complexity, huge water consumption, high production costs and large fixed capital investment. In China the fermentation industry discharges about 8 billion tons of wastewater per year, accounting for 10% of the total industrial emissions [1]. Therefore, the green sustainable development of the fermentation industry appears to be very important, which can benefit from the continuous microbial production running stably for a long period of time like the industrial chemo-process.

The co-culture systems of photo-autotrophic and heterotrophic species can play a key role in establishing the continuous fermentation mode for the biobased chemicals production. Hays et al. [2] described that model cyanobacterium *S. elongatus* PCC 7942 could co-culture with *Bacillus subtilis*, *E. coli* or *Saccharomyces cerevisiae* for a long period of time. The previous study on the co-culture of algae and bacteria suggested that the niche complementarity and functional redundancy led to high efficiency and stability of the co-culture systems in the face of biotic or abiotic disturbances [3]. The continuous and symbiotic growth was achieved and maintained for 3.5 years by co-culturing algal species *Scenedesmus obliquus*, *Scenedesmus* sp. D202, aerobic bacterial species *Bacillus* sp. D320 and *Rhodobacter sphaeroides*, and diazotrophic bacterial species *Methanobacteria* sp. D422 and *Spirulina* sp. D11 [4]. Cong et al. artificially constructed a co-culture system between *Candida tropicalis* and *S. obliquus*. Compared with the axenic culture system, the biomass and photosynthetic activity of *S. obliquus* were increased by 30.3% and 61% respectively [5]. The co-culture of *Synechocystis* PCC6803 with *Pseudomonas*-related GM41 strain resulted in eightfold increase in the cyanobacterial biomass [6]. Zhang et al. [7] reported that compared with axenic culture, co-culture of microalgae *Chlorella vulgaris* and yeast *Rhodotorula glutinis* could increase biomass by 17.3% and lipid yields by 70.9%. Shu et al. [8] enhanced CO_2_ fixation and oil production by co-culturing *Chlorella* and *S. cerevisiae*. Therefore, the co-culture systems of photo-autotrophic and heterotrophic species can contribute to the efficient and stable fermentation with high productivity and yield.

Although some co-culture systems of photo-autotrophic and heterotrophic species were successfully established, only a few efforts have been devoted to investigating the interaction mechanisms between photo-autotrophic and heterotrophic species at multi-level of RNA, protein and metabolite. The transcriptomics, proteomics and metabolomics are critical to study the interaction mechanisms of the co-culture systems. Due to the incompleteness and complementarity of these different methods, the multi-omics analysis can obtain a “panorama” of cells in the co-culture systems and demonstrate novel insights into the biological mechanisms. Amin et al. investigated the signaling and interaction between cosmopolitan phytoplankton and associated bacteria through transcriptomic and targeted metabolite analyses, which is the milestone in the analysis of the interaction between algae and bacteria. Tryptophan and indole-3-acetic acid were determined to be the key signalling molecules, which were part of a complex metabolites exchange including bacterial-excreted ammonia and diatom-excreted organosulfur molecules [9]. Therefore the methods of transcriptome, proteome and metabolome should be adopted to analyze microbial interactions in the co-culture systems, which might finally contribute to the technology breakthrough of the continuous fermentation mode.

Isoprene is an ideal raw material for synthetic rubber, and 95% of isoprene production is used for the production of synthetic rubber. With the depletion of the nonrenewable petrochemical resources and the increasing global environmental concerns, bio-isoprene will be a promising alternative towards the petroleum-isoprene. A renewable and reliable source of isoprene is imperative to economic competitiveness and national security [10]. So far, some progresses in axenic culture have been made for the biosynthesis of isoprene using various microorganisms such as *S. cerevisiae*, *Synechocystis*, *B. subtilis* and *E. coli* [11–16]. The breakthrough has been made by Genencor, which developed an *E. coli*-based system with the ability to produce > 60 g/L of isoprene [10]. However, there are no reports for the biosynthesis of isoprene by co-culture fermentation yet.

Although cyanobacteria can have the symbiosis with bacteria and fungi in natural environments, no natural symbiosis relations between cyanobacteria and heterotrophic specie *E. coli* are reported so far. The synthetic consortium of the sucrose-secreting cyanobacterium *S. elongatus* PCC 7942 and *E. coli* was constructed recently [2]. However, this novel consortium is a complete light-driven system with low cell density, which constrained the possibly wide application in the fermentation industry. In the present paper, a novel co-culture fermentation system of model microorganisms *S. elongatus* PCC 7942 and *E. coli* BL21(DE3) was established for the production of isoprene with high cell concentration. The effects of *S. elongatus* PCC 7942 on *E. coli* BL21(DE3) were also investigated using methods of omics analysis.

## Materials and methods

### Strains, plasmids and media

Strains and plasmids were listed in Table 1. *S. elongatus* PCC 7942 was generously provided by Dr. Xuefeng Lv’s laboratory [17]. BL21(DE3) was used as the host for isoprene production. The isoprene producer (IP) harboring plasmid pYJM14 with mevalonate pyrophosphate decarboxylase gene *ERG19*, mevalonate kinase gene *ERG12*, phosphomevalonate kinase gene *ERG8* and IPP isomerase gene *IDI1*, and plasmid pYJM20 with isoprene synthase gene *ispS*, HMG-CoA synthase gene *mvaS* and acetyl-CoA acetyltransferase/hydroxymethylglutaryl-CoA (HMG-CoA) reductase gene *mvaE* [18].

**Table 1:** Strains and plasmids used in this study

Strain/plasmid	Relevant genotype/property	Source/references
Strains		
*E. coli* BL21(DE3)	F^−^ *ompT hsdS*_*B*_ (*r*_*B*_^*−*^*m*_*B*_^*−*^) *gal dcm rne131*(*DE3*)	Invitrogen
*S. elongatus* PCC 7942	Wild type	[17]
Isoprene producer IP	BL21(DE3)/pYJM14 and pYJM20	[18]
Plasmids		
pYJM20	pACYCDuet-1 derivative carrying isoprene synthase gene *ispS*, HMG-CoA synthase gene *mvaS* and acetyl-CoA acetyltransferase/hydroxymethylglutaryl-CoA (HMG- CoA) reductase gene *mvaE*, T7 promoter, Cm^R^	[18]
pYJM14	pTrcHis2B derivative carrying mevalonate pyrophosphate decarboxylase gene *ERG19*, mevalonate kinase gene *ERG12*, phosphomevalonate kinase gene *ERG8* and IPP isomerase gene *IDI1*, Trc promoter, Ap^R^	[18]

*Synechococcus elongatus* PCC 7942 was propagated in BG11 medium. The isoprene producer was propagated in LB medium. The co-culture medium consisted of 0.75 g/L NaNO_3_, 0.014 g/L CaCl_2_, 0.01 g/L NaCO_3_, 0.5 mg/L Na_2_EDTA·2H_2_O, 4.9 g/L K_2_HPO_4_·3H_2_O, 1 g/L citric acid·H_2_O, 0.15 g/L ferric ammonium citrate, 1 g/L (NH_4_)_2_SO_4_, 0.21 g/L MgSO_4_, 5 g/L glucose, 3.4 μg/mL chloramphenicol, 10 μg/mL ampicillin and 0.5 ml/L trace elements [each 100 mL solution containing 0.25 g CuSO_4_·5H_2_O, 0.37 g (NH_4_)_6_Mo_7_O_24_·4H_2_O, 2.47 g H_3_BO_3_, 0.29 g ZnSO_4_·7H_2_O, 1.58 g MnCl_2_·4H_2_O].

### Co-culture for the isoprene production

The co-culture for isoprene production was performed in fed-batch manner. The seed culture of *S. elongatus* PCC 7942 was prepared in BG-11 medium for ~ 7 days at 28 ℃. The seed culture of IP-strain was prepared in LB medium supplemented with 100 μg/mL ampicillin and 34 μg/mL chloramphenicol for ~ 12 h at 37 ℃. The total 12 mL seed cultures of *S. elongatus* and IP-strain in the ratio of 1:1 or 1:4 (V/V) were used to inoculate a 250-mL bioreactor (Applikon Biotechnology, Netherland) containing 150 mL co-culture medium in light (300 μmol/m^2^/s). The initial concentrations of *S. elongatus* at the inoculation ratios of 1:1 and 1:4 (V/V) were about 8.0 × 10^6^ and 2.0 × 10^6^ cell/mL, respectively. The initial concentrations of *E. coli* at the inoculation ratios of 1:1 and 1:4 (V/V) were about 1.2 × 10^9^ and 1.8 × 10^9^ cell/mL, respectively. The chloramphenicol (3.4 μg/mL) and ampicillin (10 μg/mL) were added to the co-culture medium. The cultivation temperature was maintained at 28 ℃, the pH was controlled at 7.0 by automatic feeding concentrated glucose or 25% (w/w) KOH solution, the aeration rate was 10 vvm, and the stirring rate was maintained at 150 rpm. The cells were induced at OD_600_ 5 by the addition of 0.1 mM IPTG. The co-culture was stopped when the isoprene titer was no longer increased. The cell growth of IP-strain was assayed by plating dilution series on LB media to count colony forming units (CFU). The cell growth of *S. elongatus* PCC 7942 was measured by plating dilution series on BG-11 media to count CFU. Samples were collected for isoprene analysis at certain intervals. The co-culture for the isoprene production was carried out for three biological replicates.

### GC analysis of isoprene

1 mL of off-gas was sampled from the bioreactor by a gas syringe (PN5190-1531) and analyzed as described earlier [14] using a GC (Agilent 7890A, America) equipped with a HP-AL/S column (25 m × 320 μm × 8 μm) and FID. Nitrogen was used as carrier gas with a linear velocity of 1 mL/min. The product was characterized by comparison with standard isoprene (TCI-EP, Tokyo, Japan). The peak area was converted to isoprene concentration by a standard curve plotted with a set of known concentration of isoprene.

### RNA isolation and differential transcriptome analysis

Total RNA was extracted by the Easyspin RNA reagent (Aidlab, Beijing, China) and treated with RNase-free DNase I (Takara, Dalian, China) to remove genomic DNA contamination. RNA concentration was measured by Qubit® RNA Assay Kit in Qubit® 2.0 Flurometer (Life Technologies, CA, USA). RNA purity was checked by the NanoPhotometer® spectrophotometer (IMPLEN, CA, USA). RNA integrity was assessed using the RNA Nano 6000 Assay Kit of the Bioanalyzer 2100 system (Agilent Technologies, CA, USA). The library preparation, sequencing and data analysis were accomplished by Novogene, Inc. Differential expression analysis between two samples was carried out by the DESeq R package. Genes with an adjusted P-value < 0.05 found by DESeq were assigned as differentially expressed genes. Gene Ontology (GO) enrichment analysis of differential expression genes was performed by the GOseq R package. GO terms with corrected P-value less than 0.05 were considered to be significantly enriched. KOBAS software was employed for the statistical enrichment of differential expression genes in Kyoto Encyclopedia of Genes and Genomes (KEGG) pathways. The significant differential pathways were defined as those with an FDR value of ≤ 0.05.

### Total protein extraction and differential proteome analysis

Samples were minced individually with liquid nitrogen and lysed in lysis buffer containing 6 M Urea, 100 mM NH_4_HCO_3_ (pH 8) and 0.2% SDS, followed by 5 min of ultrasonication on ice. The lysate was centrifuged at 12,000×*g* for 15 min at 4 °C and the supernatant was transferred to a clean tube. Extracts from each sample were reduced with 2 mM DTT for 1 h at 56 °C, and subsequently alkylated with sufficient iodoacetamide for 1 h in the dark at room temperature. The samples were mixed with 4 times volume of precooled acetone and incubated at − 20 °C for at least 2 h. Samples were then centrifuged and the precipitation was collected. The pellet was dissolved by dissolution buffer containing 0.1 M triethylammonium bicarbonate (TEAB, pH 8.5) and 6 M urea after twice washing with precooled acetone. The peptide preparation, TMT labeling, HPLC fractionation, LC–MS/MS analysis and data analysis were accomplished by Novogene, Inc. The protein with at least 1 unique peptide was identified at FDR less than 1.0% on peptide and protein level, respectively. The protein quantitation results were statistically analyzed by Mann–Whitney Test, the significant ratios, defined as p < 0.05 and |log_2_FC| > 0.58 (FC > 1.5 or FC < 0.66 [fold change, FC]), were used to screen the differential expression proteins. GO analysis were conducted using the interproscan-5 program against the databases Clusters of Orthologous Groups (COG) and the non-redundant protein database (including Pfam, PRINTS, ProDom, SMART, ProSiteProfiles, PANTHER), and KEGG were employed to analyze the protein family and pathway. The enrichment pipeline was used to perform the enrichment analysis of GO and KEGG, respectively.

### Metabolites extraction, UHPLC-MS/MS analysis and data analysis

The samples (100 mg) were individually grounded with liquid nitrogen and the homogenate was resuspended with cold methanol and 0.1% formic acid by well vortexing. The samples were incubated on ice for 5 min and then were centrifuged at 15,000 rpm, 4 °C for 5 min. The supernatant was diluted to final concentration containing 60% methanol by LC–MS grade water. The samples were subsequently transferred to a fresh Eppendorf tube with 0.22 μm filter and then were centrifuged at 15,000×*g*, 4 °C for 10 min. Finally, the filtrate was injected into the UHPLC-MS/MS analysis. Metabolites extraction, UHPLC-MS/MS analysis and data analysis were accomplished by Novogene, Inc. The raw data files generated by UHPLC-MS/MS were processed using the Compound Discoverer 3.0 (CD 3.0, Thermo Fisher) to perform peak alignment, peak picking, and quantitation for each metabolite. The main parameters were set as follows: retention time tolerance, 0.2 min; actual mass tolerance, 5 ppm; signal intensity tolerance, 30%; signal/noise ratio, 3; and minimum intensity, 100,000. After that, peak intensities were normalized to the total spectral intensity. The normalized data was used to predict the molecular formula based on molecular ion peaks, additive ions and fragment ions. And then peaks were matched with ChemSpider (http://www.chemspider.com/) database and mzCloud (https://www.mzcloud.org/) to obtain the accurate qualitative and relative quantitative results. The significant ratios, defined as p < 0.05 and |log_2_FC| > 1 (FC > 2 or FC < 0.5), were used to screen the differential metabolites.

### Statistical analysis

Experiments were performed in triplicate, and the values are presented as the mean ± standard deviation. P < 0.05 was taken to indicate statistical significance.

## Results

### Establishment of the isoprene-producing co-culture system of *S. elongatus*–*E. coli*

In order to establish the isoprene-producing co-culture system of *S. elongates*–*E. coli*, the medium was designed by mixing isoprene fermentation medium [19] and BG11 culture medium at the ratio of 1:1 in volume. *S. elongatus* PCC 7942 and BL21(DE3) were introduced into the co-culture system at the inoculation ratio of 1:1 and 1:4 (*S. elongatus* PCC 7942 vs BL21(DE3), v/v) respectively. The chloramphenicol (3.4 μg/mL) and ampicillin (10 μg/mL) were added to the co-culture medium to reduce the plasmids loss of the engineered *E. coli* and increase the stability of the heterogeneous mevalonate (MVA) pathway genes. Although the low levels of chloramphenicol and ampicillin were not fatal for the cyanobacteria [20, 21], the antibiotics addition completely inhibited the growth of *S. elongatus* PCC 7942 in the axenic culture and only the axenic culture of BL21(DE3) was used as the control.

**Fig. 1:**
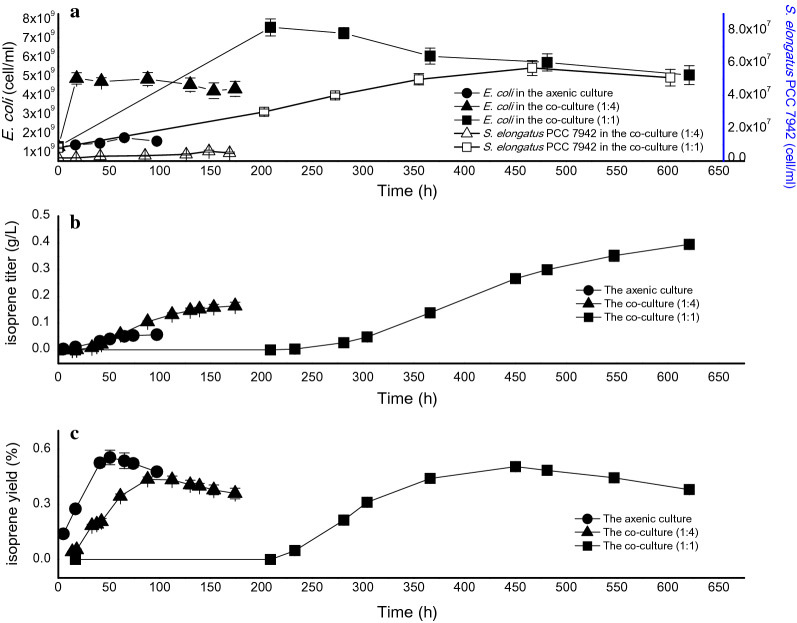
Time courses of biomass, isoprene titer and yield in the axenic culture and co-culture. The axenic culture of BL21(DE3) is the control. *S. elongatus* PCC 7942 and BL21(DE3) were introduced into the co-culture system at the inoculation ratio of 1:4 and 1:1 respectively. The circles, triangles and squares indicate the control, the co-culture1 (the inoculation ratio of 1:4) and co-culture2 (the inoculation ratio of 1:1), respectively

The time courses of biomass, isoprene titer and yield in the axenic culture and co-cultures at the different inoculation ratios were determined respectively. As shown in Fig. 1a, the cell concentration of BL21(DE3) in the co-culture was higher than the control, indicating that the presence of *S. elongatus* PCC 7942 promoted the growth of BL21(DE3). The improved growth of *S. elongatus* PCC 7942 in the co-culture suggested that the inhibition of antibiotics on *S. elongatus* PCC 7942 was released by BL21(DE3) in the co-culture system. As shown in Fig. 1b, by increasing the inoculation ratio from 1:4 in co-culture1 to 1:1 in co-culture2, the isoprene fermentation period of time was extended from 100 to 400 h. Compared to the axenic culture of BL21(DE3), the isoprene titer in co-culture was increased sevenfold to 0.4 g/L. The longer fermentation time and higher isoprene titer in the co-culture process indicated that the presence of *S. elongatus* PCC 7942 promoted the isoprene synthesis. As shown in Fig. 1c, the maximum yield of isoprene was all close to 0.5% during the processes of co-culture and axenic culture. Therefore the novel co-culture system of *S. elongates*–*E. coli* was preliminarily established.

### Differential omics analysis of *E. coli* BL21(DE3) between the co-culture system and the control

In order to study the cellular response of *E. coli* BL21(DE3) to the addition of *S. elongatus* PCC 7942, the samples (EC1, EC2 and EC3) of co-culture2 for the differential omics analysis were taken at induction time point 18 h, and the early and late periods of isoprene production (250 h and 500 h), respectively. The samples (E1) of axenic culture of BL21(DE3) were taken at 18 h as the control. Because the antibiotics addition completely inhibited the growth of *S. elongatus* PCC 7942 in the axenic culture, the differential omics analysis was not performed for the *S. elongatus* PCC 7942.

The differential profiles of gene expression, proteins and metabolites in *E. coli* BL21(DE3) were identified by pairwise comparisons of E1, EC1, EC2 and EC3 (Table 2). In pairs of EC1 vs. E1, 374 genes were differentially expressed, including 212 up-regulated genes and 162 down-regulated genes. And 359 proteins were differentially expressed, including 210 up-regulated proteins and 149 down-regulated proteins. There were 83 differential metabolites (55 up-regulations and 28 down-regulations) in positive mode. There were 105 differential metabolites (78 up-regulations and 27 down-regulations) in negative mode. In pairs of EC2 vs. EC1, 856 genes were differentially expressed, including 397 up-regulated genes and 459 down-regulated genes. Seventy proteins were differentially expressed, including 52 up-regulated proteins and 18 down-regulated proteins. There were 141 differential metabolites (110 up-regulations and 31 down-regulations) in positive mode. There were 97 different metabolites (64 up-regulations and 33 down-regulations) in negative mode. In pairs of EC3 vs. EC2, 224 genes were differentially expressed, including 54 up-regulated genes and 170 down-regulated genes. Twenty proteins were differentially expressed, including 17 up-regulated proteins and 3 down-regulated proteins. There were 181 differential metabolites (43 up-regulations and 138 down-regulations). There were 105 different metabolites (42 up-regulations and 63 down-regulations) in negative mode.

**Table 2:** The differential profiles of gene expression, proteins and metabolites in *E. coli* BL21(DE3) in pairwise comparisons of E1, EC1, EC2 and EC3

Samples	EC1 vs. E1		EC2 vs. EC1		EC3 vs. EC2	
	Negative	Positive	Negative	Positive	Negative	Positive
mRNAs						
Total	374		856		224	
Up	212		397		54	
Down	162		459		170	
Proteins						
Total	359		70		20	
Up	210		52		17	
Down	149		18		3	
Metabolites						
Total	105	83	97	141	105	181
Up	78	55	64	110	42	43
Down	27	28	33	31	63	138

### Functional analysis of differential transcriptome, proteome and metabolome

**Fig. 2:**
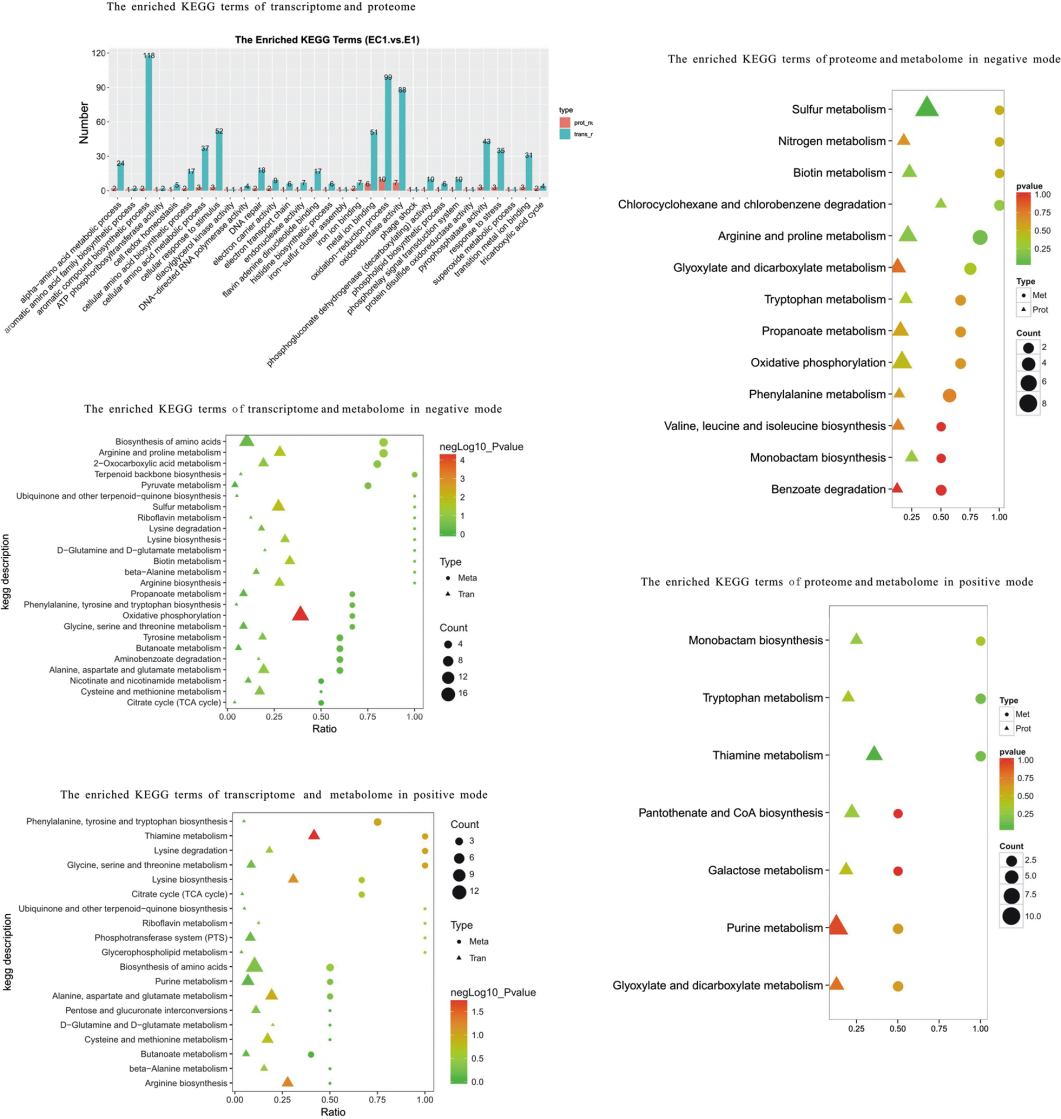
Functional analysis of differential transcriptome, proteome and metabolome in pairs of EC1 vs. E1. The KEGG enrichment analysis was carried out for the differentially expressed proteins (genes) in proteome and transcriptome (red represents up-regulation and blue represents down-regulation). The KEGG enrichment analysis was carried out for the differentially expressed profiles in proteome and metabolome. The circles and triangles represent the differential metabolites and proteins in the corresponding pathway respectively. The KEGG enrichment analysis was also carried out for the differentially expressed profiles in transcriptome and metabolome. The circles and triangles represent the differential metabolites and transcripts in the corresponding pathway respectively. “Count” is the number of genes, metabolites or proteins enriched in the pathway. “Ratio” is the ratio of the number of differential genes, metabolites or proteins to the number of genes, metabolites or proteins annotated in the pathway. The colors of circles and triangles represent the p-value of the hyper-geometric test

As shown in Fig. 2, KEGG enrichment analysis of transcriptome and proteome suggested that the flagellar assembly and thiamine metabolism in EC1 were significantly down-regulated while ribosome was up-regulated compared to E1. Dozens of differential transcripts and proteins were assigned to the tryptophan metabolism, sulfur metabolism, pyrimidine and purine metabolisms, arginine and proline metabolisms, branched chain amino acid metabolisms, pantothenate and CoA metabolism, biotin metabolisms, lipoic acid metabolism, homologous recombination, DNA replication and base excision repair. GO enrichment analysis of transcriptome and proteome also showed that compared to E1, flagella-dependent cell motility was significantly down-regulated, and the processes and molecular functions involved with ribosome were mainly up-regulated in EC1 (Additional file 1: Fig. S1). Both KEGG and GO enrichment analyses indicated that in EC1 the flagellum-dependent motility of *E. coli* strains decreased significantly, but the metabolic activity increased significantly.

As shown in Fig. 2, KEGG enrichment analysis of transcriptome and metabolome suggested that compared to the control E1, thiamine metabolism, arginine synthesis and metabolism, sulfur metabolism, lysine synthesis and degradation, TCA, ubiquinone synthesis, riboflavin metabolism, tyrosine metabolism, glutamic acid metabolism, purine metabolism and aromatic compound degradation were significantly changed in EC1.

As shown in Fig. 2, KEGG enrichment analysis of proteome and metabolome showed that compared to the control E1, tryptophan metabolism, thiamine metabolism, sulfur metabolism, arginine metabolism, purine metabolism, pantothenic acid and coenzyme A metabolism, biotin metabolism, phenylalanine metabolism, benzoic acid metabolism, glyoxylate metabolism and branched chain amino acid synthesis significantly varied in EC1.

**Fig. 3:**
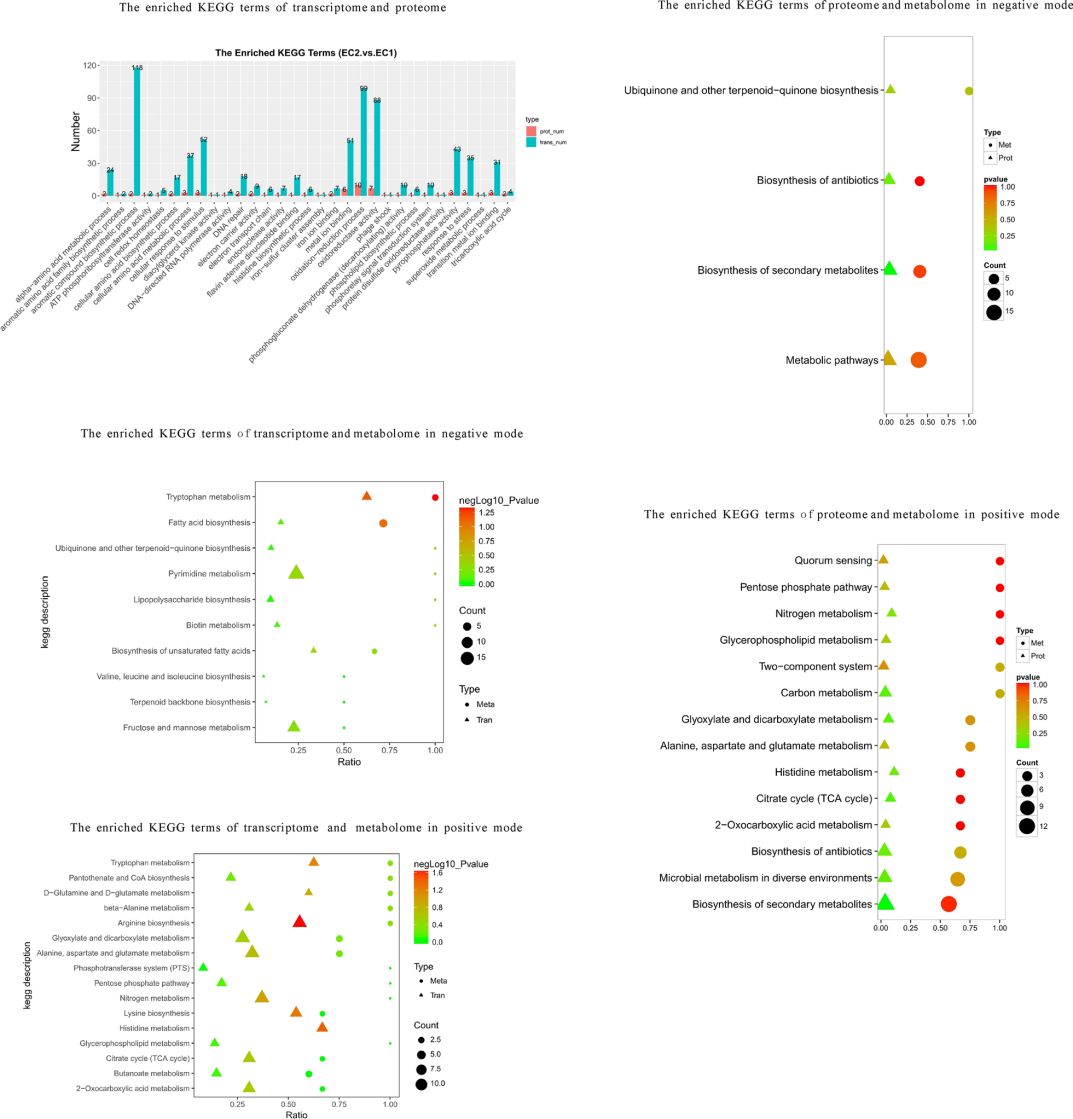
Functional analysis of differential transcriptome, proteome and metabolome in pairs of EC2 vs. EC1. The KEGG enrichment analysis was carried out for the differentially expressed proteins (genes) in proteome and transcriptome (red represents up-regulation and blue represents down-regulation). The KEGG enrichment analysis was carried out for the differentially expressed profiles in proteome and metabolome. The circles and triangles represent the differential metabolites and proteins in the corresponding pathway respectively. The KEGG enrichment analysis was also carried out for the differentially expressed profiles in transcriptome and metabolome. The circles and triangles represent the differential metabolites and transcripts in the corresponding pathway respectively. “Count” is the number of genes, metabolites or proteins enriched in the pathway. “Ratio” is the ratio of the number of differential genes, metabolites or proteins to the number of genes, metabolites or proteins annotated in the pathway. The colors of circles and triangles represent the p-value of the hyper-geometric test

As shown in Fig. 3, KEGG enrichment analysis of transcriptome and proteome suggested that the folate synthesis, tryptophan metabolism and histamine metabolism, homologous recombination, and base excision repair took significant changes in EC2 compared to EC1. The GO enrichment analysis of transcriptome and proteome showed that folate synthesis also changed significantly (Additional file 1: Fig. S2). KEGG and GO enrichment analyses of transcriptome showed that compared to the control EC1, ribosomes and the metabolism and degradation of fatty acids were significantly changed in EC2.

As shown in Fig. 3, KEGG enrichment analysis of transcriptome and metabolome showed that compared to the control EC1, the fatty acid synthesis, the synthesis of unsaturated fatty acids, tryptophan metabolism, arginine synthesis and metabolism, ubiquinone synthesis, phenylalanine metabolism, the degradation of aromatic compounds, pyrimidine metabolism, pantothenic acid metabolism, biotin metabolism, and CoA metabolism, TCA significantly varied in EC2.

As shown in Fig. 3, KEGG enrichment analysis of proteome and metabolome showed that compared to the control EC1, the histidine metabolism, quorum sensing, TCA, PPP, ubiquinone synthesis, glutamate metabolism and glyoxylic acid had significant changes in EC2.

**Fig. 4:**
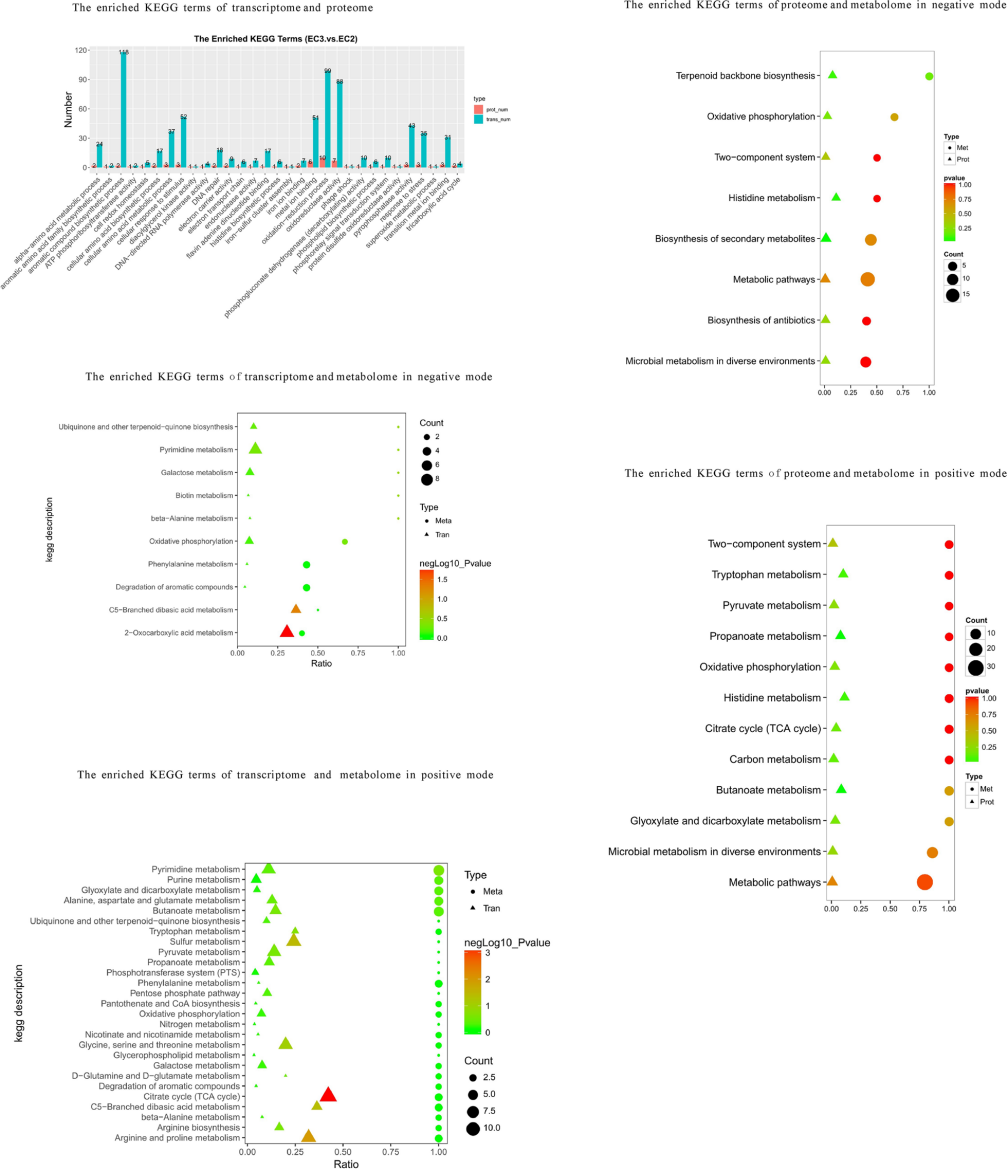
Functional analysis of differential transcriptome, proteome and metabolome in pairs of EC3 vs. EC2. The KEGG enrichment analysis was carried out for the differentially expressed proteins (genes) in proteome and transcriptome (red represents up-regulation and blue represents down-regulation). The KEGG enrichment analysis was carried out for the differentially expressed profiles in proteome and metabolome. The circles and triangles represent the differential metabolites and proteins in the corresponding pathway respectively. The KEGG enrichment analysis was also carried out for the differentially expressed profiles in transcriptome and metabolome. The circles and triangles represent the differential metabolites and transcripts in the corresponding pathway respectively. “Count” is the number of genes, metabolites or proteins enriched in the pathway. “Ratio” is the ratio of the number of differential genes, metabolites or proteins to the number of genes, metabolites or proteins annotated in the pathway. The colors of circles and triangles represent the p-value of the hyper-geometric test

As shown in Fig. 4, KEGG enrichment analysis of transcriptome and proteome suggested that the biofilm formation, TCA cycle, lysine synthesis and degradation, fatty acid synthesis, metabolism and degradation were significantly changed in EC3 compared to EC2. The GO enrichment analysis of transcriptome and proteome showed that the redox process and redox enzymes were mainly up-regulated at the protein level while were mainly down-regulated at the transcription level in EC3 compared to EC2, indicating that the metabolic activity of *E. coli* strains in EC3 was changing from prosperity to decline (Additional file 1: Fig. S3).

As shown in Fig. 4, KEGG enrichment analysis of transcriptome and metabolome showed that compared to EC2, the synthesis of ubiquinone, arginine and proline metabolisms, tyrosine metabolism, phenylalanine metabolism, aromatic compounds degradation, TCA, glyoxylic acid metabolism, purine and pyrimidine metabolisms, biotin metabolism, lysine synthesis and degradation, sulfur metabolism, tryptophan metabolism, fatty acid synthesis and degradation significantly varied in EC3.

As shown in Fig. 4, KEGG enrichment analysis of proteome and metabolome showed that compared to EC2, fatty acid metabolism and degradation, terpene synthesis, histidine metabolism, oxidative phosphorylation, two-component system, TCA, tryptophan metabolism, pyruvate metabolism, propionic acid metabolism, glyoxylate metabolism had significant changes in EC3.

### Correlation analysis of differential transcriptome, proteome and metabolome

**Fig. 5:**
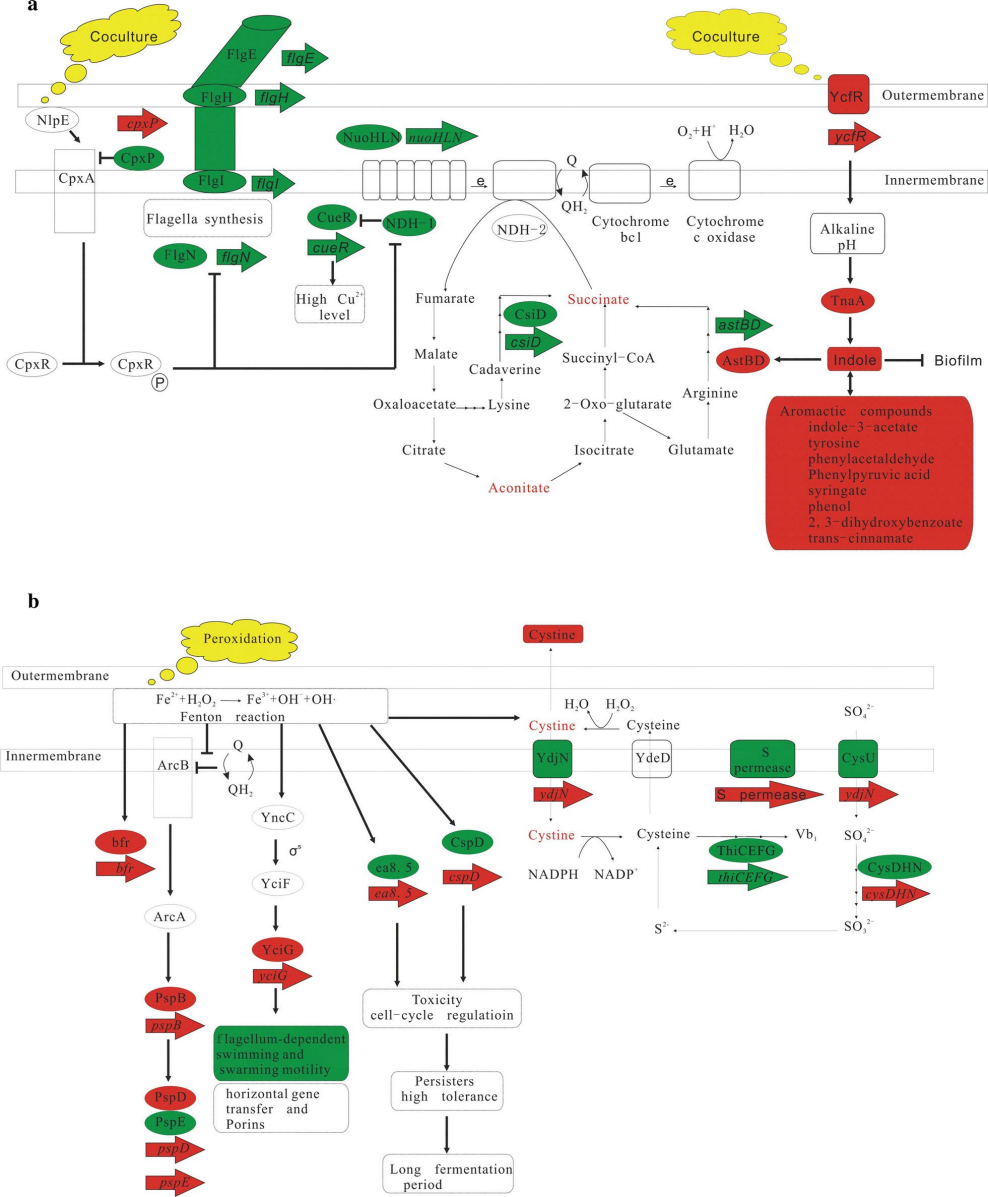
Correlation analysis of differential transcriptome, proteome and metabolome in pairs of EC1 vs. E1. The red represents up-regulation and the green represents down-regulation

**Fig. 6:**
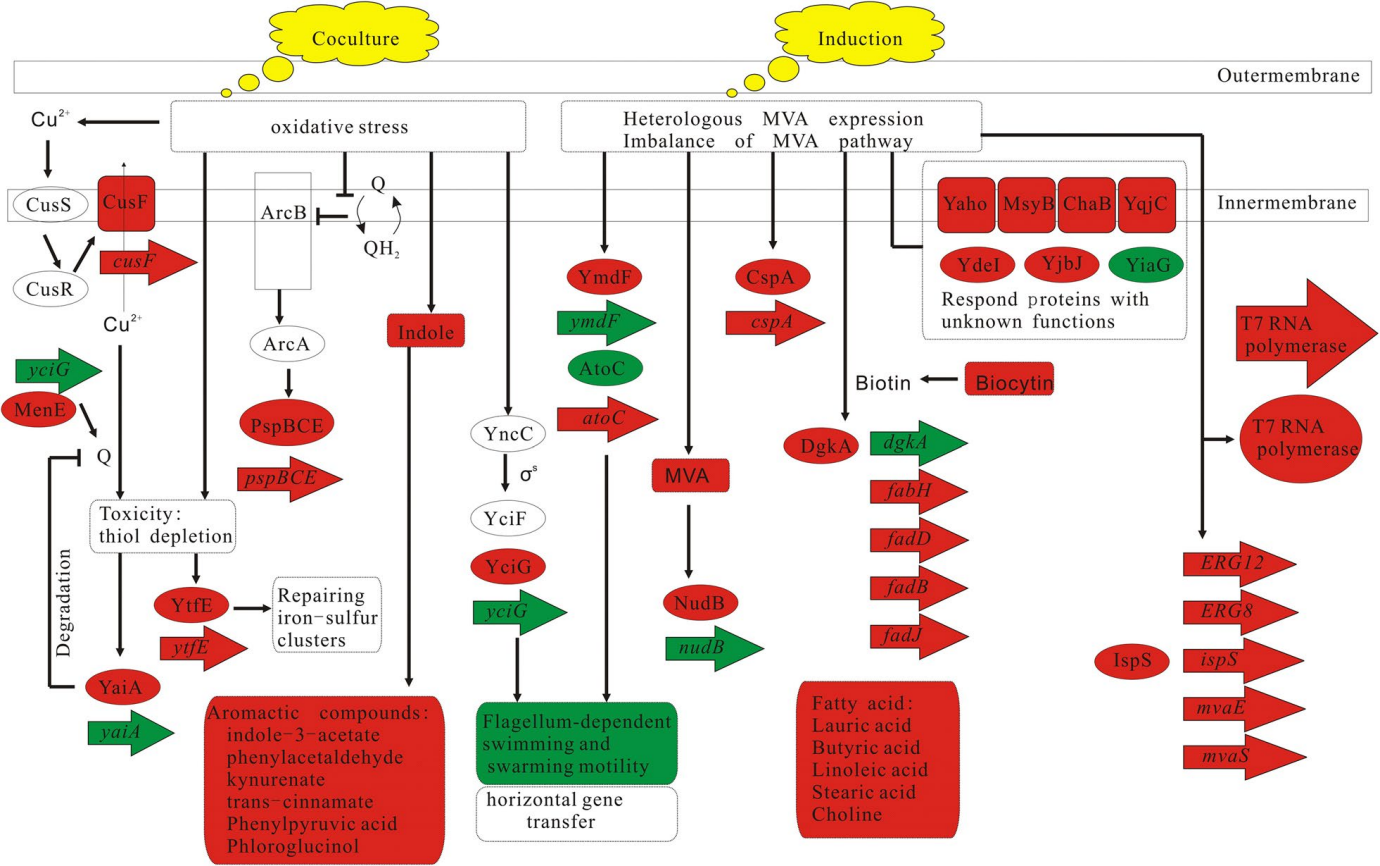
Correlation analysis of differential transcriptome, proteome and metabolome in pairs of EC2 vs. EC1. The red represents up-regulation and the green represents down-regulation

The response of BL21(DE3) to the *S. elongatus* PCC 7942 was investigated through the omics correlation analysis. A series of changes at the levels of transcription, protein, and metabolism were detected (Figs. 5 and 6). Firstly, BFR for binding ferrous ion was up-regulated and the YtfE for repairing iron-sulfur clusters was up-regulated. The thiol cysteine was converted to cystine. The downstream thiamine synthesis was subsequently down-regulated. Secondly, the toxic proteins ea8.5 and CspD and the phage shock proteins PspB, PspD and PspE were up-regulated to cope with the oxidative pressure. Thirdly, the flagella synthesis and assembly (FlagE, FlagI, FlagH, FlagN) were decreased. The protein YciG, relevant to the motility of cells and horizontal gene transfer, was upregulated. Fourthly, the expression of NADH: ubiquinone oxidoreductase I in the respiratory chain of *E. coli* strains was inhibited by CpxR due to the oxidative pressure or the physical contact of *S. elongatus* PCC 7942, which led to the up-regulation of CusF for strengthening the efflux of Cu^2+^ and the up-regulation of YaiF for the degradation of ubiquinone. MenE was also up-regulated to make up the loss of the ubiquinone after the release of the Cu^2+^ toxicity. The expressions of menE and yaiA were all subsequently decreased in EC2 compared to EC1. Fifthly, many aromatic compounds such as indole and ubiquinone increased, sharing the common precursor chorismate in the biosynthesis pathway. The stress-responsing protein YcfR was upregulated to increase the indole production for the inhibition of the biofilm formation and the strenghthening of arginine catabolism. The differential expressions of proteins AstBD implied that the glutamate could be recycled through arginine catabolism.

**Fig. 7:**
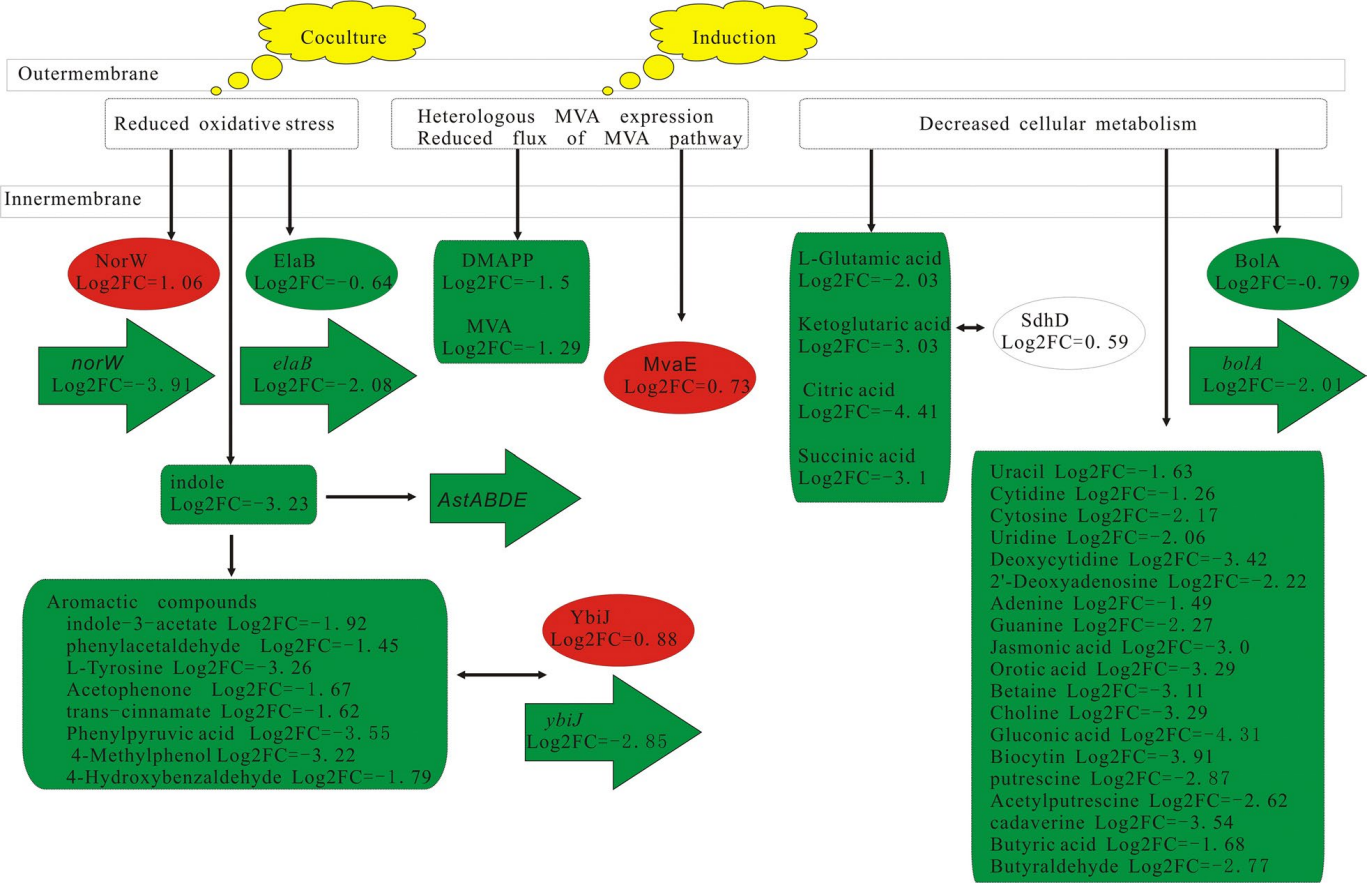
Correlation analysis of differential transcriptome, proteome and metabolome in pairs of EC3 vs. EC2. The red represents up-regulation and the green represents down-regulation

As shown in Figs. 6 and 7, the overexpression of protein puts pressure on the host after the addition of IPTG, leading to up-regulation of cold-shock protein. Both the mRNA and protein of the isoprene synthase were detected to be up-regulated in EC2 and kept unchanged in EC3. In contrast, MvaE were gradually up-regulated and kept increasing in EC3. The intermediate metabolites (MVA and DMAPP) in MVA pathway were also observed to be accumulated. The pyrophosphohydrolase NudB was up-regulated to release the accumulation of toxic DMAPP. In addition, the fatty acid biosynthesis was increased.

## Discussion

### Response of *E. coli* BL21(DE3) to *S. elongatus* PCC 7942 in co-culture system

The response of BL21(DE3) to *S. elongatus* PCC 7942 in this co-culture system was actually triggered by the oxidative pressure from photosynthesis. The inter-species cross-protection from oxidative pressure has been noted for photoautotrophic cyanobacteria and ‘helper’ heterotrophs in many natural symbiotic systems [22–24]. Cyanobacteria are often inhibited by reactive oxygen species (ROS) produced from the imbalance between light collection and light use during the process of photosynthesis. The inhibition of oxidative pressure on cyanobacteria can be released by the ‘helper’ heterotrophic bacteria through eliminating ROS. This inter-species interaction has also recently been proved in artificial co-culture systems of *S. elongatus* [25, 26]. However, the growth of heterotrophs can be inhibited by ROS when the heterotrophs were exposed to high densities of cyanobacteria in the light [2, 27]. The influences of oxidative pressure from photosynthesis on cellular response of ‘helper’ heterotrophic bacteria have not been investigated in details previously. Despite some studies on the catalase expression of the heterotrophs [28, 29], much work remains to be done in order to understand this helping phenomenon from the heterotroph. Considering that the heterotrophic *E. coli* is essential for the isoprene production in the co-culture system, the response of *E. coli* to the oxidative pressure from photosynthesis was systematically investigated.

In the present paper, we discovered that the *S. elongatus* PCC 7942 led to oxidative pressure on the *E. coli* strains through the Fenton reaction, which triggered a series of changes at the levels of transcription, protein, and metabolism. All the changes were linked with one another at different spatial and temporal scale. Firstly, in order to stop the Fenton reaction, BFR was up-regulated to decrease the ferrous ion concentration and the thiol cysteine was converted to cystine to reduce H_2_O_2_. The downstream thiamine synthesis was subsequently down-regulated to support the production of cysteine. The cystine importer was also up-regulated to support the conversion of cystine to cysteine. The Fe–S proteins on the cell membrane were damaged by the Fenton reaction and the YtfE was up-regulated for the repair of damaged proteins. Secondly, the toxic proteins ea8.5 and CspD were upregulated to change the cell cycle and improve the cell tolerance, which could contribute to the long-lasting fermentation process. Thirdly, the phage shock proteins PspB, PspD and PspE were up-regulated to cope with the oxidative pressure. Fourthly, the flagella synthesis and assembly were decreased. The protein YciG, relevant to the motility of cells and horizontal gene transfer, was upregulated. Thus the swimming and swarming mobility of *E. coli* BL21(DE3) strains were weakened. Fifthly, the expression of NADH: ubiquinone oxidoreductase I in the respiratory chain of *E. coli* strains was inhibited by CpxR due to the oxidative pressure. The NADH consumption through the oxidative phosphorylation was reduced and the NADH pool was rebalanced through the glutmate biosynthesis and metabolism. The differential proteins AstBD implied that the glutamate could be recycled through arginine catabolism. High level of Cu^2+^ coexisting with ubiquinone is toxic to the strains because of the thiol depletion in EC2, which triggered the up-regulation of YtfE for repairing iron–sulfur clusters, the up-regulation of CusF for strengthening the efflux of Cu^2+^ and the up-regulation of YaiF for the degradation of ubiquinone. MenE was also up-regulated to make up the loss of the ubiquinone after the release of the Cu^2+^ toxicity. In order to keep the balance of ubiquinone pool, the expressions of menE and yaiA were all decreased in EC2 compared to EC1. Sixthly, the stress-responsing protein YcfR was upregulated to increase the indole production for the inhibition of the biofilm formation and the strenghthening of arginine catabolism. Finally, the aromatic compounds such as indole and ubiquinone increased, sharing the common precursor chorismate in the biosynthesis pathway. Therefore, the regulation of photosynthesis is the key influencing factor of co-culture.

### Cross-feeding and competition between *S. elongatus* and *E. coli*

Both cross-feeding and competition between *S. elongatus* and *E. coli* are the beneficial interactions for the fermentation system. The inter-species cross-feeding is an important class of interactions in the co-culture system of *S. elongatus* and *E. coli*. *S. elongatus* supplies photosynthetically-derived oxygen and carbon to *E. coli*. The extracellular polymeric substances (EPS) can be used to sustain the growth of *E. coli* [30]. *E. coli* can improve the growth of *S. elongatus* by scavenging wastes (e.g. oxygen and ROS) and providing key metabolites (e.g. carbon dioxide) [23, 31–33]. In addition to the cross-feeding, the competition also existed in the co-culture system of *S. elongatus* and *E. coli*. On one hand, the introduction of *S. elongatus* may be beneficial to the fermentation system through the competition similar to the catfish effect. The oxidative pressure from *S. elongatus* triggered a series of changes in *E. coli* as described previously. The oxidative stress mitigation pathways might contribute to the long-lasting fermentation process. On the other hand, *E. coli* can counter the oxidative pressure from *S. elongatus* by secreting toxic amino acids (histidine, phenylalanine, lysine) to inhibit *S. elongatus*, which could contribute to the balance between the two species in the co-culture system. The histidine was detected to be upregulated (log_2_FC = 1.78). The significant upregulation of *pheA* (log_2_FC = 1.07) in the biosynthesis pathway of the phenylalanine in *E. coli* was observed. The upregulation (log_2_FC = 3.51) of phenylacetaldehyde, the degradation metabolite of phenylalanine, was also detected. The significant upregulation of *lysC* (log_2_FC = 1.33) and *dapB* (log_2_FC = 1.82) in the biosynthesis pathway of lysine in *E. coli* was observed. Considering that cyanobacteria have a low utilization ability of exogenous amino acids, and the exogenous amino acids (histidine, phenylalanine, lysine) are toxic to cyanobacteria [34, 35], the up-regulated toxic amino acids (histidine, phenylalanine, lysine) could limit the growth of *S. elongatus*. The Previous study has also shown that the addition of yeast extract or amino acids in the culture medium can inhibit the growth of *S. elongatus* [26].

### Effects of induction on the isoprene-producing *E. coli* strain

The introduction of heterologous metabolic pathways into the chassis cell often results in low productivity and high cellular stress, especially for those with unbalanced assembled pathways [14, 36, 37]. Therefore, the effects of IPTG induction on the *E. coli* strains were investigated. The overexpression of protein puts pressure on the host, leading to up-regulation of cold-shock protein. Similar to the results described by George et al. the intermediate metabolites (MVA and DMAPP) in MVA pathway were also observed to be accumulated. The difference was that George et al. [36] described the physiological response to high IPP accumulation with severe growth inhibition, while we discussed the response without the growth inhibition.

The accumulation of intermediate metabolites had great effects on the endogenous metabolism of *E. coli* strains. Firstly, the pyrophosphohydrolase NudB was up-regulated to release the accumulation of toxic DMAPP. Secondly, the fatty acid biosynthesis was increased as described previously, which showed that isoprenoids-producing *E. coli* strains suffered from the disturbance of fatty acid biosynthesis [38]. It might be the differential expressions of key enzymes in MVA pathway that resulted in the imbalance of the isoprene biosynthesis pathway and the accumulation of the intermediate metabolites. Both the mRNA and protein of the isoprene synthase were detected to be up-regulated in EC2 and kept unchanged in EC3. In contrast, MvaE were gradually up-regulated and kept increasing in EC3.

### Potential of the co-culture system of *S. elongatus *PCC 7942 and *E. coli* BL21(DE3) for isoprene production

Although this co-culture system had a long fermentation period of time for isoprene production, the isoprene titer was relatively lower than the previous reports. Yang et al. [18] reported that the isoprene production of 6.3 g/L was achieved in a fed-batch culture by *E. coli* using hybrid MVA pathway. Liu et al. [14] established a two-step process with the final isoprene production of 11.0 g/L by dividing the MVA pathway into the upstream process from sugar to MVA, and the downstream process from MVA to isoprene. Genencor developed an *E. coli*-based production system with the ability to produce > 60 g/L of isoprene [10]. In spite of this great gap, the isoprene production in the co-culture could be further improved by process control optimizations and strain improvements according to the fundamental rules discovered in the present paper.

Process control optimizations can improve the performance of co-culture systems of *S. elongates*–*E. coli* by monitoring key indexes of processes, regulating the light availability, essential nutrient supplementation, induction mode and using the isoprene-producing engineered *S. elongatus* PCC 7942 in place of the wild type. Firstly, the monitoring of co-culture process is very important for process control optimizations. According to the omics analysis, the accumulation of intermediate metabolites (MVA, DMAPP, amino acids, etc.) and the ratio of microalgae and bacteria should be the key monitoring indexes. Secondly, the light availability may influence the growth of cyanobacteria in any algal systems through photosynthesis [39]. The light availability may further influence the cross-feeding of metabolites between *S. elongatus* PCC 7942 and *E. coli* BL21(DE3) including oxygen and carbon dioxide through photosynthesis and respiration. The light availability may also affect the oxidative pressure on the *E. coli* strains due to the photosynthesis. Therefore, the optimal light conditions can finally contribute to the high cell density co-culture with proper ratio of *S. elongatus* PCC 7942 and *E. coli* BL21(DE3). Thirdly, the glucose and nitrogen limitation, the induction mode and betaine supplementation were found to be the key factors for isoprene production through the systematical optimization of fermentation conditions [14]. Finally, the photosynthetic isoprene production by cyanobacteria from CO_2_ has been proved to be feasible previously [15, 16]. So *S. elongatus* PCC 7942 could be engineered for isoprene production to improve the performance of this co-culture system. The inhibition of antibiotics can also be completely released by replacing the wild type with the isoprene-producing engineered *S. elongatus* PCC 7942 resistant to the antibiotics.

When performing strain improvements, the rational design of *E. coli* strain should be derived from the omics correlation analysis of this co-culture system. Firstly, the *E. coli* strains could be engineered to be more suitable for the co-culture systems by coping with the oxidative pressure from photosynthesis. The engineered *E. coli* strains with improved oxidative stress mitigation pathways could contribute to the stability and high efficiency of synthetic consortia. It is interesting that the natural consortia have similar mechanisms to mitigate oxidative stress of reactive oxygen species (ROS) or elevated local oxygen levels by the heterotrophs, i.e., catalase-dependent ROS scavenging by *Prochlorococcus* [24, 29]. Secondly, the *E. coli* strains could be improved to reduce the accumulation of MVA and isopentenyl diphosphate (IPP) by employing the balanced MVA pathway. The balanced biosynthetic pathway can keep the key enzyme stable, avoid the disturbance to overall cell system and maximize metabolic flux to the isoprene. The careful managements of MVA and IPP levels have been described previously in details through the evaluation on the promoters, ribosome binding sites, copy number of genes, key enzymes, novel IPP-bypass pathway and novel MVA-mediated pathway [10, 37, 40–42]. All of the strategies can be applied to balance MVA for the isoprene production.

### Co-culture systems of the model algae and bacteria without natural symbiotic relation and the common interaction mechanisms

The study on the co-culture system of the model algae and bacteria without natural symbiotic relation is of significance to reveal the common interactions between photo-autotrophic and heterotrophic species. The natural consortia such as the unicyanobacterial consortium (UCC) are usually pervasive and robust. In UCC the cyanobacterium supplies photosynthetically-derived oxygen and carbon to the heterotrophs, while the heterotrophs can improve the growth of cyanobacterium by scavenging wastes and providing key metabolites [43, 44]. Although the natural symbiotic relation is often regarded as the prerequisite for the establishment of co-culture, the artificial synthetic consortia have been constructed by engineering the microbes interacting with one another through molecular signals or metabolic intermediates (i.e., complementary auxotrophs, quorum sensing) [45–48]. Furthermore, some attempts have recently been devoted to the design and construction of co-culture systems composed of model photo-autotrophic and heterotrophic species without natural symbiotic relation. Cong et al. artificially constructed a co-culture system between the model microorganisms *C. tropicalis* and *S. obliquus* [5]. Zhang et al. [7] described a co-culture system of *C. vulgaris* and oleaginous yeast *Rhodotorula glutinis*. Shu et al. [8] enhanced CO_2_ fixation and oil production by co-culturing the model Chlorella and *S. cerevisiae*. Hays et al. [2] designed the synthetic consortia composed of model cyanobacterium and heterotrophic species such as *B. subtilis*, *E. coli* and *S. cerevisiae*. These studies including the co-culture of *S. elongatus* PCC 7942 and *E. coli* BL21(DE3) indicated that there might be a unspecific interaction between photo-autotrophic and heterotrophic species. More studies on the co-culture systems of the model photo-autotrophic and heterotrophic species are required to reveal the common interaction mechanism.

### Bottlenecks of microbial culture in industry and the potential of co-culture systems

The current culture methods for different types of microorganisms including microalgae, yeast, mold and bacterium have bottlenecks that need to be solved in the commercial production of bio-based chemicals. First of all, the open pond systems are often employed for the commercial-scale cultivation of microalgae, whereas the low biomass and poor process stability are the bottlenecks for the cultivation of microalgae in open ponds [49–52]. The concentration of microalgae in the open ponds is only 0.1–0.5 g/L under the condition of large-scale culture, which is too low to achieve efficient carbon fixation and chemical production. In addition, the sustained microalgae cultivation in the open ponds is successful only for a few algal species such as spirulina, chlorella and dunaliella due to the biotic and abiotic pressures [52, 53]. Secondly, although the concentration of microalgae can be significantly increased in the closed photo-bioreactors compared to the open ponds, there are still problems that need to be solved urgently such as the toxicity of high dissolved oxygen and low fixation efficiency of carbon dioxide [5, 49, 54]. The concentration of dissolved oxygen can be increased to the high level of 35 mg/L by photosynthesis in the closed systems, especially for the high density culture [5, 54]. The high concentration of dissolved oxygen will strongly inhibit the growth of microalgae and the fixation of carbon dioxide [5]. Thirdly, the high cell density of heterotrophic microorganisms can be easily achieved in fermentation industry from many renewable sources (such as starch, sugar, wood fiber material, etc.). Many fuels and platform chemicals such as ethanol, isoprene, antibiotics, amino acids, organic acid, vitamins and steroids, can be produced by microbial fermentation in industry [55]. However, the majority of microbial fermentations are non-continuous processes with high water consumption and wastewater discharge. During the conventional fermentation process, a large amount of by-products are accumulated and lots of alkaline liquor is introduced in the fermentation broth, which result in the high salinity and COD, and finally make the fermentation process non-continuous.

In short, non-continuous fermentation process, and the instability and low efficient microalgae culture are the current problems of microbial culture in industry. The commercial production of biobased chemicals can greatly benefit from the continuous microbial culture with high efficiency and robustness like the industrial chemo-process. In recent, some new efforts have been focused on the co-culture systems of photo-autotrophic and heterotrophic species [2, 5, 7, 25, 26, 56], which are expected to be the breakthrough in solving the bottlenecks of microbial culture, while the current progress is far from enough. Microalgae is often associated with bacteria in natural symbioses or long-term laboratory cultures [56]. The co-culture systems of phototrophs and heterotrophs can make full use of the complementary features of multispecies to achieve micro-ecological balance for the continuous, stable and efficient culture. For examples, the aerobic fermentation cultures demand a high and continuous oxygen supply [57–59]. The significant oxygen limitations can occur due to the incomplete micromixing in large-scale fermenters [60]. The aeration cost is high on the order of $ 60 million per year and up to 26% of the overall utility cost in a plant that processes 2000 t of dry biomass per day [37]. The improved oxygen supply and reduced aeration cost can be potentially achieved by the cross-feeding of oxygen and carbon dioxide between phototrophs and heterotrophs through photosynthesis and respiration. Furthermore, the photosynthesis can increase the pH value of the culture system, while the heterotrophic metabolism can decrease the pH [49, 54, 61, 62]. So the co-culture of phototrophs and heterotrophs is also beneficial to maintain the acid–base balance of the culture system. In addition, the cross-feeding of key metabolites (e.g. vitamin B_12_, iron-binding siderophores or indole acetic acid) is very important for the continuous microbial culture [23, 32, 33]. Therefore, the co-culture systems of photo-autotrophic and heterotrophic species can play important roles in establishing the continuous fermentation mode for the bio-based chemicals production.

## Conclusions

In the present paper, the isoprene-producing co-culture system of *S. elongates*–*E. coli* was established in this study and the differential omics analysis was carried out for this novel system. Many changes were discovered to be linked with one another at different spatial and temporal scales due to the oxidative pressure on *E. coli* from *S. elongatus* through the Fenton reaction. According to the omics analysis, the performance of this co-culture system can be further improved. This study on the co-culture of the algae and bacteria without natural symbiotic relation is of significance to reveal the common interactions between photo-autotrophic and heterotrophic species.

## Supplementary Information

**Additional file 1: Figure S1.** Functional analysis of differential transcriptome and proteome in pairs of EC1 vs. E1. The GO enrichment analysis was carried out for the differentially expressed proteins (genes) in proteome and transcriptome (red represents up-regulation and blue represents down-regulation). **Figure S2.** Functional analysis of differential transcriptome and proteome in pairs of EC2 vs. EC1. The GO enrichment analysis was carried out for the differentially expressed proteins (genes) in proteome and transcriptome (red represents up-regulation and blue represents down-regulation). **Figure S3.** Functional analysis of differential transcriptome and proteome in pairs of EC3 vs. EC2. The GO enrichment analysis was carried out for the differentially expressed proteins (genes) in proteome and transcriptome (red represents up-regulation and blue represents down-regulation).

## Data Availability

The datasets used and analyzed during the current study are available from the corresponding author on reasonable request.

## References

[1] Zhang S (2011). Current status and prospects of technology development in fermentation engineering. Biotechnol Bus..

[2] Hays SG, Yan LLW, Silver PA, Ducat DC (2017). Synthetic photosynthetic consortia define interactions leading to robustness and photoproduction. J Biol Eng.

[3] Hamilton CE (2014). Exploring the utilization of complex algal communities to address algal pond crash and increase annual biomass production for algal biofuels.

[4] Dhamwichukorn S. Method for enhanced sustainable production of algal bio-products, comprising use of symbiotic diazotroph-attenuated stress co-cultivation. Organization WIP ed., vol. WO2011022229A22011.

[5] Wang R, Tian Y, Xue S, Zhang D, Zhang Q, Wu X, Kong D, Cong W (2016). Enhanced microalgal biomass and lipid production via co-culture of *Scenedesmus obliquus* and *Candida tropicalis* in an autotrophic system. J Chem Technol Biotechnol.

[6] Abed RMM (2010). Interaction between cyanobacteria and aerobic heterotrophic bacteria in the degradation of hydrocarbons. Int Biodeterior Biodegrad.

[7] Zhang Z, Ji H, Gong G, Xu Z, Tan T (2014). Synergistic effects of oleaginous yeast *Rhodotorula glutinis* and microalga *Chlorella vulgaris* for enhancement of biomass and lipid yields. Bioresour Technol.

[8] Shu C-H, Tsai C-C, Chen K-Y, Liao W-H, Huang H-C (2013). Enhancing high quality oil accumulation and carbon dioxide fixation by a mixed culture of *Chlorella* sp. and *Saccharomyces cerevisiae*. J Taiwan Inst Chem Eng.

[9] Amin SA, Hmelo LR, Tol HMV, Durham BP, Carlson LT, Heal KR, Morales RL, Berthiaume CT, Parker MS, Djunaedi B (2015). Interaction and signalling between a cosmopolitan phytoplankton and associated bacteria. Nature.

[10] Whited GM, Feher FJ, Benko DA, Cervin MA, Chotani GK, McAuliffe JC, LaDuca RJ, Ben-Shoshan EA, Sanford KJ (2010). Development of a gas-phase bioprocess for isoprene-monomer production using metabolic pathway engineering. Ind Biotechnol.

[11] Lv X, Xu H, Yu H (2013). Significantly enhanced production of isoprene by ordered coexpression of genes dxs, dxr, and idi in *Escherichia coli*. Appl Microbiol Biotechnol.

[12] Liu H, Sun Y, Ramos KRM, Nisola GM, Valdehuesa KNG, Lee WK, Park SJ, Chung W-J (2013). Combination of Entner–Doudoroff pathway with MEP increases isoprene production in engineered *Escherichia coli*. PLoS ONE.

[13] Yang C, Gao X, Jiang Y, Sun B, Gao F, Yang S (2016). Synergy between methylerythritol phosphate pathway and mevalonate pathway for isoprene production in *Escherichia coli*. Metab Eng.

[14] Liu H, Cheng T, Zou H, Zhang H, Xu X, Sun C, Aboulnaga E, Cheng Z, Zhao G, Xian M (2017). High titer mevalonate fermentation and its feeding as a building block for isoprenoids (isoprene and sabinene) production in engineered *Escherichia coli*. Process Biochem.

[15] Gao X, Gao F, Liu D, Zhang H, Nie X, Yang C (2016). Engineering the methylerythritol phosphate pathway in cyanobacteria for photosynthetic isoprene production from CO_2_. Energy Environ Sci.

[16] Lindberg P, Park S, Melis A (2010). Engineering a platform for photosynthetic isoprene production in cyanobacteria, using Synechocystis as the model organism. Metab Eng.

[17] Du W, Liang F, Duan Y, Tan X, Lu X (2013). Exploring the photosynthetic production capacity of sucrose by cyanobacteria. Metab Eng.

[18] Yang J, Xian M, Su S, Zhao G, Nie Q, Jiang X, Zheng Y, Liu W (2012). Enhancing production of bio-isoprene using hybrid MVA pathway and isoprene synthase in *E. coli*. PLoS ONE.

[19] Zhao Y, Yang J, Qin B, Li Y, Sun Y, Su S, Xian M (2011). Biosynthesis of isoprene in *Escherichia coli* via methylerythritol phosphate (MEP) pathway. Appl Microbiol Biotechnol.

[20] Liu X, Sheng J, Curtiss R (2011). Fatty acid production in genetically modified cyanobacteria. Proc Natl Acad Sci.

[21] Li S, Zhi Y, Li P, Wang F, Chen W (2018). Effects of chloramphenicol on carboxysome number and cellular abundance of CcmK2 in *Synechocystis* sp. PCC 6803. J Huazhong Agric Univ..

[22] Venn AA, Loram JE, Douglas AE (2008). Photosynthetic symbioses in animals. J Exp Bot.

[23] Morris JJ, Johnson ZI, Szul MJ, Keller M, Zinser ER (2011). Dependence of the cyanobacterium *Prochlorococcus* on hydrogen peroxide scavenging microbes for growth at the ocean’s surface. PLoS ONE.

[24] Morris JJ, Kirkegaard R, Szul MJ, Johnson ZI, Zinser ER (2008). Facilitation of robust growth of *Prochlorococcus* colonies and dilute liquid cultures by “helper” heterotrophic bacteria. Appl Environ Microbiol.

[25] Zhang L, Chen L, Diao J, Song X, Shi M, Zhang W (2020). Construction and analysis of an artificial consortium based on the fast-growing cyanobacterium *Synechococcus elongatus* UTEX 2973 to produce the platform chemical 3-hydroxypropionic acid from CO_2_. Biotechnol Biofuels.

[26] Li T, Li C-T, Butler K, Hays SG, Guarnieri MT, Oyler GA, Betenbaugh MJ (2017). Mimicking lichens: incorporation of yeast strains together with sucrose-secreting cyanobacteria improves survival, growth, ROS removal, and lipid production in a stable mutualistic co-culture production platform. Biotechnol Biofuels.

[27] Imlay JA (2013). The molecular mechanisms and physiological consequences of oxidative stress: lessons from a model bacterium. Nat Rev Microbiol.

[28] Hennon GMM, Morris JJ, Haley ST, Zinser ER, Durrant AR, Entwistle E, Dokland T, Dyhrman ST (2018). The impact of elevated CO_2_ on *Prochlorococcus* and microbial interactions with ‘helper’ bacterium Alteromonas. ISME J.

[29] Beliaev AS, Romine MF, Serres M, Bernstein HC, Linggi BE, Markillie LM, Isern NG, Chrisler WB, Kucek LA, Hill EA (2014). Inference of interactions in cyanobacterial–heterotrophic co-cultures via transcriptome sequencing. ISME J.

[30] Villa F, Pitts B, Lauchnor E, Cappitelli F, Stewart PS (2015). Development of a laboratory model of a phototroph–heterotroph mixed-species biofilm at the stone/air interface. Front Microbiol.

[31] Ma M, Eaton JW (1992). Multicellular oxidant defense in unicellular organisms. Proc Natl Acad Sci.

[32] Croft MT, Lawrence AD, Raux-Deery E, Warren MJ, Smith AG (2005). Algae acquire vitamin B12 through a symbiotic relationship with bacteria. Nature.

[33] de Bashan LE, Antoun H, Bashan Y (2008). Involvement of indole-3-acetic acid produced by the growth-promoting bacterium *Azospirillum* spp. in promoting growth of *Chlorella vulgaris*. J Phycol.

[34] Labarre J, Thuriaux P, Chauvat F (1987). Genetic analysis of amino acid transport in the facultatively heterotrophic cyanobacterium *Synechocystis* sp. strain 6803. J Bacteriol.

[35] Hall GC, Jensen RA (1980). Enzymological basis for growth inhibition by l-phenylalanine in the cyanobacterium *Synechocystis* sp. 29108. J Bacteriol.

[36] George KW, Thompson MG, Kim J, Baidoo EEK, Wang G, Benites VT, Petzold CJ, Chan LJG, Yilmaz S, Turhanen P (2018). Integrated analysis of isopentenyl pyrophosphate (IPP) toxicity in isoprenoid-producing *Escherichia coli*. Metab Eng.

[37] Kang A, George KW, Wang G, Baidoo E, Keasling JD, Lee TS (2016). Isopentenyl diphosphate (IPP)-bypass mevalonate pathways for isopentenol production. Metab Eng.

[38] Kizer L, Pitera DJ, Pfleger BF, Keasling JD (2008). Application of functional genomics to pathway optimization for increased isoprenoid production. Appl Environ Microbiol.

[39] Xue S, Su Z, Cong W (2011). Growth of *Spirulina platensis* enhanced under intermittent illumination. J Biotechnol.

[40] Yang J, Nie Q, Liu H, Xian M, Liu H (2016). A novel MVA-mediated pathway for isoprene production in engineered *E. coli*. BMC Biotechnol.

[41] Zhang Q, Lou Y, Yang J, Wang J, Feng J, Zhao Y, Wang L, Huang X, Fu Q, Ye M (2019). Integrated multiomic analysis reveals comprehensive tumour heterogeneity and novel immunophenotypic classification in hepatocellular carcinomas. Gut.

[42] Cheng T, Liu H, Zou H, Chen N, Shi M, Xie C, Zhao G, Xian M (2017). Enzymatic process optimization for the in vitro production of isoprene from mevalonate. Microb Cell Fact.

[43] Paerl HW, Pinckney JL (1996). A mini-review of microbial consortia: their roles in aquatic production and biogeochemical cycling. Microb Ecol.

[44] Paerl HW, Pinckney JL, Steppe TF (2000). Cyanobacterial—bacterial mat consortia: examining the functional unit of microbial survival and growth in extreme environments. Environ Microbiol.

[45] Zhang H, Wang X (2016). Modular co-culture engineering, a new approach for metabolic engineering. Metab Eng.

[46] Chen Y (2011). Development and application of co-culture for ethanol production by co-fermentation of glucose and xylose: a systematic review. J Ind Microbiol Biotechnol.

[47] Hays SG, Patrick WG, Ziesack M, Oxman N, Silver PA (2015). Better together: engineering and application of microbial symbioses. Curr Opin Biotechnol.

[48] Scott SR, Hasty J (2016). Quorum sensing communication modules for microbial consortia. ACS Synth Biol.

[49] Chisti Y (2007). Biodiesel from microalgae. Biotechnol Adv.

[50] Carney LT, Wilkenfeld JS, Lane PD, Solberg OD, Fuqua ZB, Cornelius NG, Gillespie S, Williams KP, Samocha TM, Lane TW (2016). Pond crash forensics: presumptive identification of pond crash agents by next generation sequencing in replicate raceway mass cultures of *Nannochloropsis salina*. Algal Res.

[51] Smith VH, Crews T (2014). Applying ecological principles of crop cultivation in large-scale algal biomass production. Algal Res.

[52] Wang H, Zhang W, Chen L, Wang J, Liu T (2013). The contamination and control of biological pollutants in mass cultivation of microalgae. Bioresour Technol.

[53] Parmar A, Singh NK, Pandey A, Gnansounou E, Madamwar D (2011). Cyanobacteria and microalgae: a positive prospect for biofuels. Biores Technol.

[54] Kumar A, Ergas S, Yuan X, Sahu A, Zhang Q, Dewulf J, Malcata FX, van Langenhove H (2010). Enhanced CO_2_ fixation and biofuel production via microalgae: recent developments and future directions. Trends Biotechnol.

[55] Lee SY, Kim HU, Chae TU, Cho JS, Kim JW, Shin JH, Kim DI, Ko Y-S, Jang WD, Jang Y-S (2019). A comprehensive metabolic map for production of bio-based chemicals. Nat Catal.

[56] Han J, Zhang L, Wang S, Yang G, Zhao L, Pan K (2016). Co-culturing bacteria and microalgae in organic carbon containing medium. J Biol Res Thessalon.

[57] Ouyang P, Wang H, Hajnal I, Wu Q, Guo Y, Chen G-Q (2018). Increasing oxygen availability for improving poly(3-hydroxybutyrate) production by Halomonas. Metab Eng.

[58] Lara AR, Knabben I, Regestein L, Sassi J, Caspeta L, Ramírez OT, Büchs J (2011). Comparison of oxygen enriched air vs. pressure cultivations to increase oxygen transfer and to scale-up plasmid DNA production fermentations. Eng Life Sci.

[59] Carcamo M, Saa PA, Torres J, Torres S, Mandujano P, Correa JRP, Agosin E (2014). Effective dissolved oxygen control strategy for high-cell-density cultures. IEEE Latin Am Trans.

[60] Garcia JR, Cha HJ, Rao G, Marten MR, Bentley WE (2009). Microbial nar-GFP cell sensors reveal oxygen limitations in highly agitated and aerated laboratory-scale fermentors. Microb Cell Fact.

[61] Wang Y, Ding H, Du P, Gan R, Ye Q (2005). Production of phoA promoter-controlled human epidermal growth factor in fed-batch cultures of *Escherichia coli* YK537 (pAET-8). Process Biochem.

[62] Chung W-J, Huang C-L, Gong H-Y, Ou T-Y, Hsu J-L, Hu S-Y (2015). Recombinant production of biologically active giant grouper (*Epinephelus lanceolatus*) growth hormone from inclusion bodies of *Escherichia coli* by fed-batch culture. Protein Expr Purif.

