# Supplementary material for: Study on the isoprene-producing co-culture system of Synechococcus elongates–Escherichia coli through omics analysis
Source: Microb Cell Fact. 2021 Jan 7;20:6. doi: 10.1186/s12934-020-01498-8 (PMC7791884; doi:10.1186/s12934-020-01498-8)
Supplement: Supplementary file 1 — Additional file 1: Figure S1. Functional analysis of differential transcriptome and proteome in pairs of EC1 vs. E1. The GO enrichment analysis was carried out for the differentially expressed proteins (genes) in proteome and transcriptome (red represents up-regulation and blue represents down-regulation). Figure S2. Functional analysis of differential transcriptome and proteome in pairs of EC2 vs. EC1. The GO enrichment analysis was carried out for the differentially expressed proteins (genes) in proteome and transcriptome (red represents up-regulation and blue represents down-regulation). Figure S3. Functional analysis of differential transcriptome and proteome in pairs of EC3 vs. EC2. The GO enrichment analysis was carried out for the differentially expressed proteins (genes) in proteome and transcriptome (red represents up-regulation and blue represents down-regulation). [file 12934_2020_1498_MOESM1_ESM.doc]

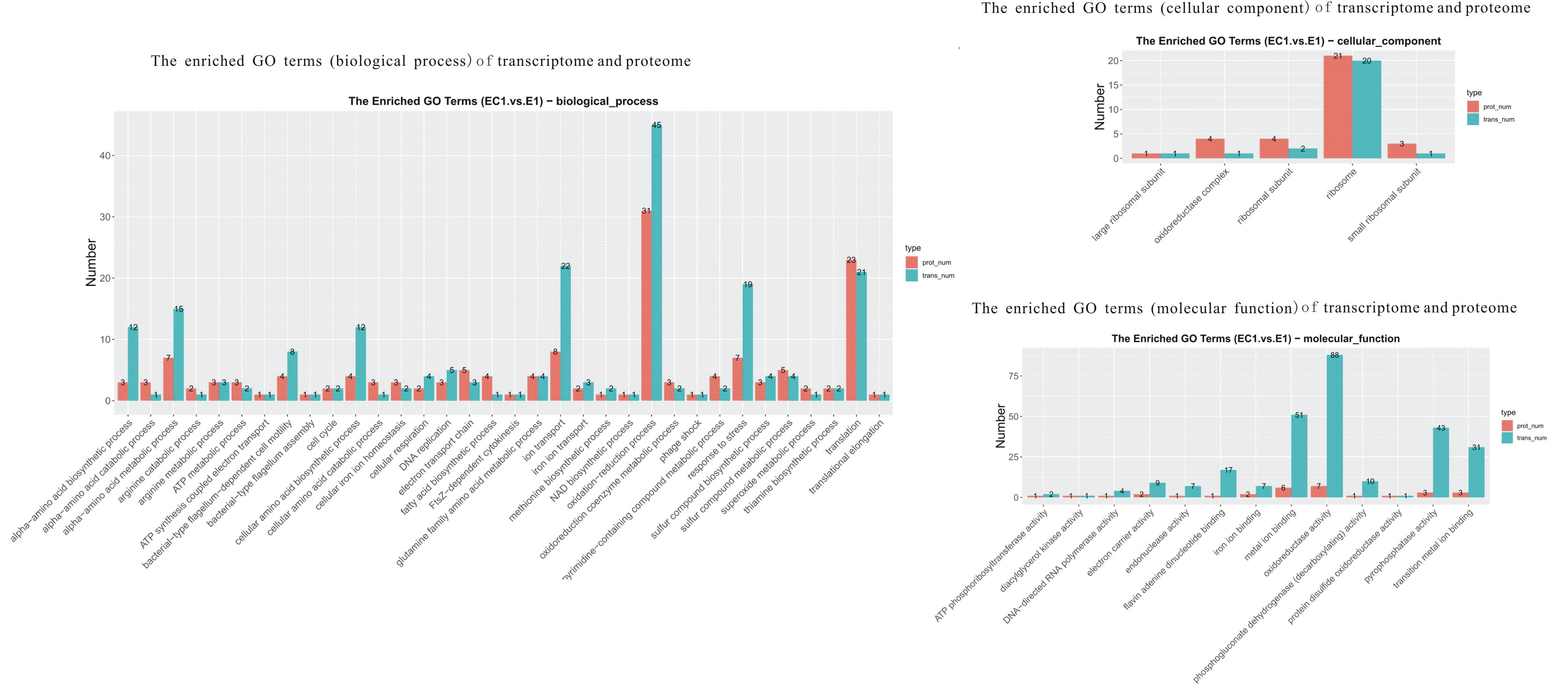


Fig. S1 Functional analysis of differential transcriptome and proteome in pairs of EC1 vs. E1. The GO enrichment analysis was carried out for the differentially expressed proteins (genes) in proteome and transcriptome (Red represents up-regulation and blue represents down-regulation).


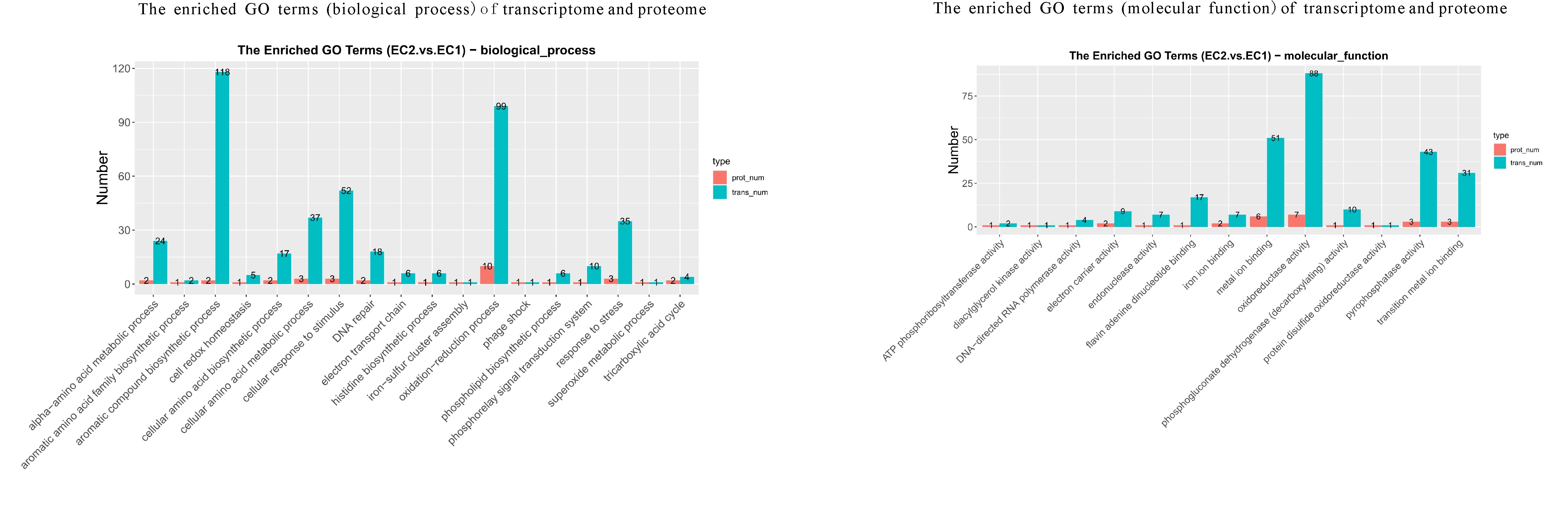


Fig. S2 Functional analysis of differential transcriptome and proteome in pairs of EC2 vs. EC1. The GO enrichment analysis was carried out for the differentially expressed proteins (genes) in proteome and transcriptome (Red represents up-regulation and blue represents down-regulation).


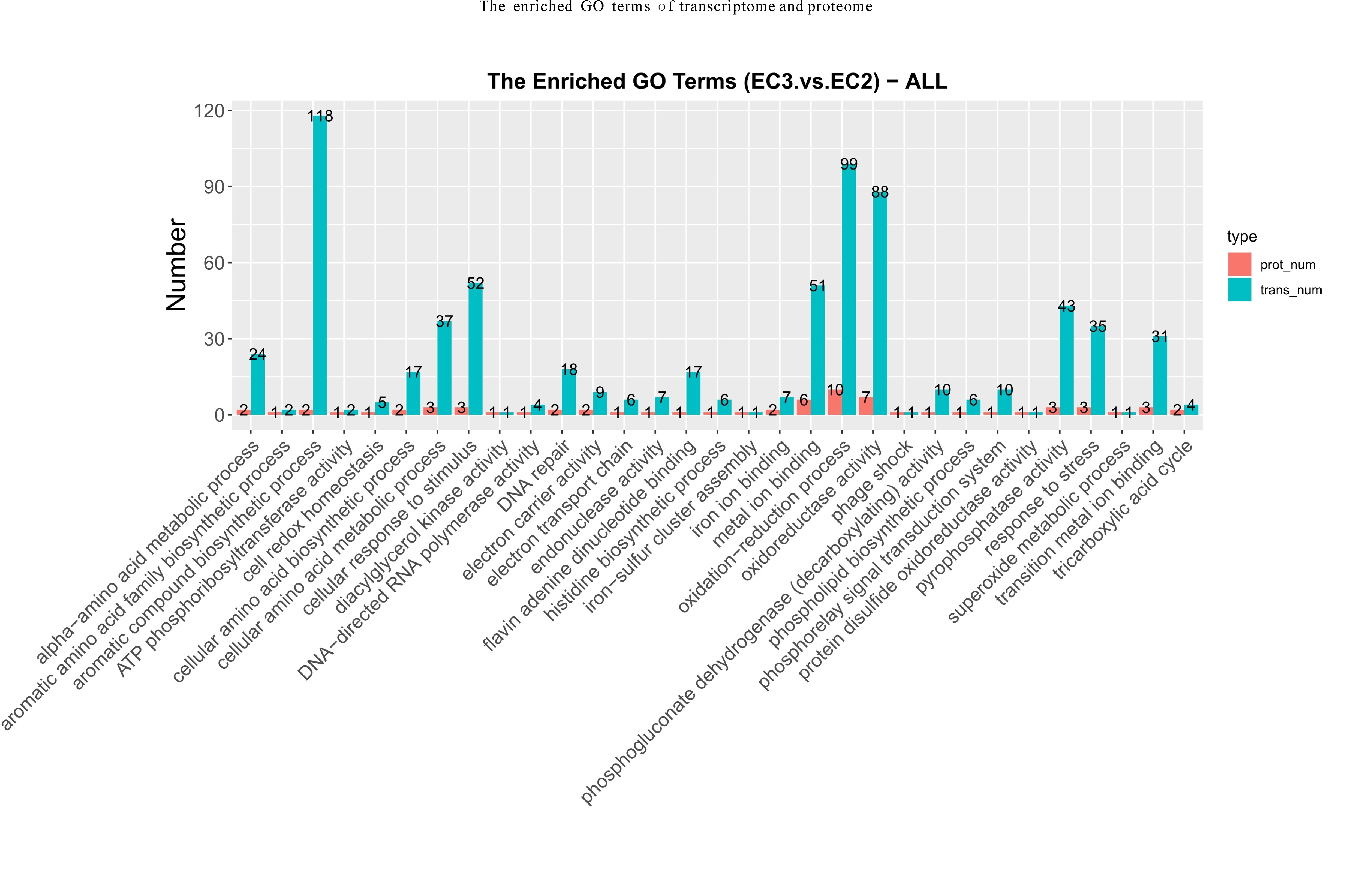


Fig. S3 Functional analysis of differential transcriptome and proteome in pairs of EC3 vs. EC2. The GO enrichment analysis was carried out for the differentially expressed proteins (genes) in proteome and transcriptome (Red represents up-regulation and blue represents down-regulation).
